# Synthetic car dataset for vehicle detection: Integrating aerial and satellite imagery

**DOI:** 10.1016/j.dib.2024.110105

**Published:** 2024-02-06

**Authors:** Mihaela Orić, Vlatko Galić, Filip Novoselnik

**Affiliations:** Protostar Labs, HR31551 Belišće, Croatia

**Keywords:** Synthetic dataset, Blender, Car detection, Computer vision, Machine learning

## Abstract

Vehicle detection is a very important aspect of computer vision application to aerial and satellite imagery, facilitating activities such as instance counting, velocity estimation, traffic predictions, etc. The feasibility of accurate vehicle detection often depends on limited training datasets, requiring a lot of manual work in collection and annotation tasks. Furthermore, there are no known publicly available datasets. Our aim was to construct a pipeline for synthetic dataset generation from aerial imagery and 3D models in Blender software. The dataset generation pipeline consists of seven steps and results in a wished number of images with bounding boxes in YOLO and coco formats. This synthetic dataset has been produced following the steps described in this pipeline. It consists of 5000 2048 × 2048 images with cars inserted into the roads and highways at the images without cars from all over the world. We believe that this dataset and the respective pipeline might be of great importance for vehicle detection, facilitating the customizability of the models to specific needs and context.

Specifications Table

**Table:** 

Subject	Computer Science / Computer Vision and Pattern Recognition
Specific subject area	Car/object detection on aerial images
Data format	Raw
Type of data	Images, text files (annotations), json file (annotations)
Data collection	Google maps areas with no cars on the road were selected and imported to Blender. By using the information about road points on the map, 3D models of cars were positioned on the roads. Colors of the cars were manipulated to cover the real representation of cars on roads. Sun position in Blender is modified to fit the geo location of the area to ensure the correct length and angle of the shadows. The resolution and blur of cars are modified to fit the resolution of the map. Images are rendered and annotations with bounding boxes are written.
Data source location	Data were collected via OpenStreetMap at various locations containing features such as roads and highways.
Data accessibility	Full dataset containing 5000 images is hosted at Zenodo repo. Repository name: Zenodo Data identification number: (or DOI or persistent identifier) 10.5281/zenodo.10276846 Direct URL to data: https://zenodo.org/records/10276846 Instructions for accessing these data: Download the zip file.

## 1 Value of the Data

• This dataset contains 5000 images of cars taken from aerial viewpoint with the resolution similar to satellite images which are resource hungry to collect. Using this dataset speeds up and simplifies the data collection and annotation process.
• Data is modeled to fit the real look of cars from high altitudes while keeping in mind not only the resolution of cars but also the color representation. Data has a wide range of areas with different looks (different saturation, B/W balance, contrast), geographical areas (towns, villages, islands), and types of roads (highway, two-lane roads, pavement, dirt roads).
• Using a dataset in this scale and size allows researchers more flexibility in model training and parameter testing and is suitable for deep learning. The scale of this dataset also allows researchers to experiment with combining real data and synthetic data with the option to make the synthetic data percentage very high. The dataset creation pipeline is not limited to the size and allows the recreation of the dataset on a larger or smaller scale.
• This dataset is fit for object detection training with cars being the main focus. However, researchers can use the data creation steps in this article to create datasets that cover rare use cases or contain rare vehicles. This gives the community the freedom to fit this pipeline to the specific needs of their research.
• Models trained on these data can be used on non real-time images, for example on satellite imagery. Detecting cars on satellite images taken at different points in time can help track the change in average traffic count for some area for a longer period in time. This can be used for various statistical analysis purposes.
• Using it on real-time data gathered from aerial vehicles (e.g., drones and helicopters) can allow for traffic detection and analysis. Images with real-time car detections can help civil engineers find critical points on roads where traffic jams are often. It can also be used for surveillance purposes to detect illegal traffic on surfaces where no traffic is allowed.

## 2 Background

Car detection in a common object detection use case. The lack of known freely available car detection datasets was the main motivation behind our research. We needed a dataset for our research where we trained a YOLO object detection model that detects cars on aerial imagery. In the future research, that model will be deployed on an edge device for car detection on drones or other UAVs. The process of collecting and annotating images of cars gets more difficult when the altitude of the camera that takes photos gets higher. On larger heights, the resolution of cars on the land gets lower making it more difficult to separate cars from the background when manually labeling the images. Furthermore, sometimes the number of cars on images taken at such heights is very high which slows down the annotation process drastically. There is a drastic room for improvement in the process of annotation with automated pipelines for data creation and annotation. Using the synthetic data in this way reduces the manual labor needed for the image annotation process. This allows for creation of large datasets that can be used in deep learning. Moreover, automated pipelines like the one used in this work reduces the possibility of errors in the annotation process. There are also cases where using real data is legally challenging, such as using images where humans are visible and their identity needs to be protected, whilst synthetic images do not have this problem. On the other hand, the domain gap between real and synthetic images can sometimes be too big. To ensure the usability of this synthetic dataset, images with cars are made to be as similar to real images as possible.

## 3 Data Description

The dataset presented in this article contains synthetic images of cars placed on roads and is structured in a single folder with images, a single folder with YOLO (*You Only Look Once*) bounding box annotations, and a single JSON file with coco annotations. The images folder contains 5000 Blender renders of scenes from maps with cars placed on the roads. Images are in PNG format with a resolution of 2048 × 2048 pixels. Images are labeled from “0.png” to “4999.png”. There are 10 different car 3D models that represent different car types that are most common on roads.

Labels folder contains text files with YOLO annotations. Each image in the images folder has its corresponding text file in the labels folder. For example, “0.txt” contains the annotations of the “0.png” image. If there is an image with no cars on it, its corresponding text file is empty but does exist. Each line in those text files refers to one car visible in the image. The first character in those lines will always be 0 and represent the car class. However, this character would change if new classes are added. label.json contains bounding boxes in coco format. Bounding boxes are in standard non-rotated format.

Alongside the zipped dataset of 5000 images, there is one smaller zip that contains 50 annotated images. The smaller dataset serves as a sample and allows for fast downloading and testing.

## 4 Experimental Design, Materials and Methods

Free and open-source 3D computer graphics software Blender [1] was used as the base program for the creation of this dataset. Its embedded Python interpreter allows every step in this pipeline to be automated. Every action that can be done in the graphical interface of Blender can also be done through Python. The process of dataset creation is described below:

### 4.1 Map selection and import

Scenes in Blender were created by importing maps using BlenderGis [2]. BlenderGis is a Blender add-on used for importing satellite maps and other map data, such as geometry of the surface, buildings, information about the map, into the scene. It can be connected to multiple map services. The basemap service for this dataset was Google Maps. Each scene in Blender was created manually by choosing areas with no cars visible and saving those map sections as base planes in the scenes. Information about the roads and land use for that map part were imported through BlenderGis as well. By using BlenderGis, each scene was automatically georeferenced (has longitude and latitude metadata). This is useful for other steps in the pipeline, which will be discussed below. Treating the map as a plane in Blender makes it affected by light and other scene properties. This means not all parts of the map will be lighted equally and the look of the colors will not match the original color. To avoid disrupting the real look of the map, it is shaded with an emission shader, and the color transform is changed to standard. This makes the map not absorb any light or shadows in the scene, but emit its real color in standard color space.

Areas without cars are manually searched for. Diverse areas were picked - suburban areas, villages, wilderness areas, highways, islands, etc. Since there are parts where roads are covered by trees, some cars were placed on top of them. Counterintuitively, this does not create an issue but rather increases the quality of the dataset since cars in real life can be found on green surfaces and not just on plain roads.

### 4.2 Adding cars to images

**Image 1:**
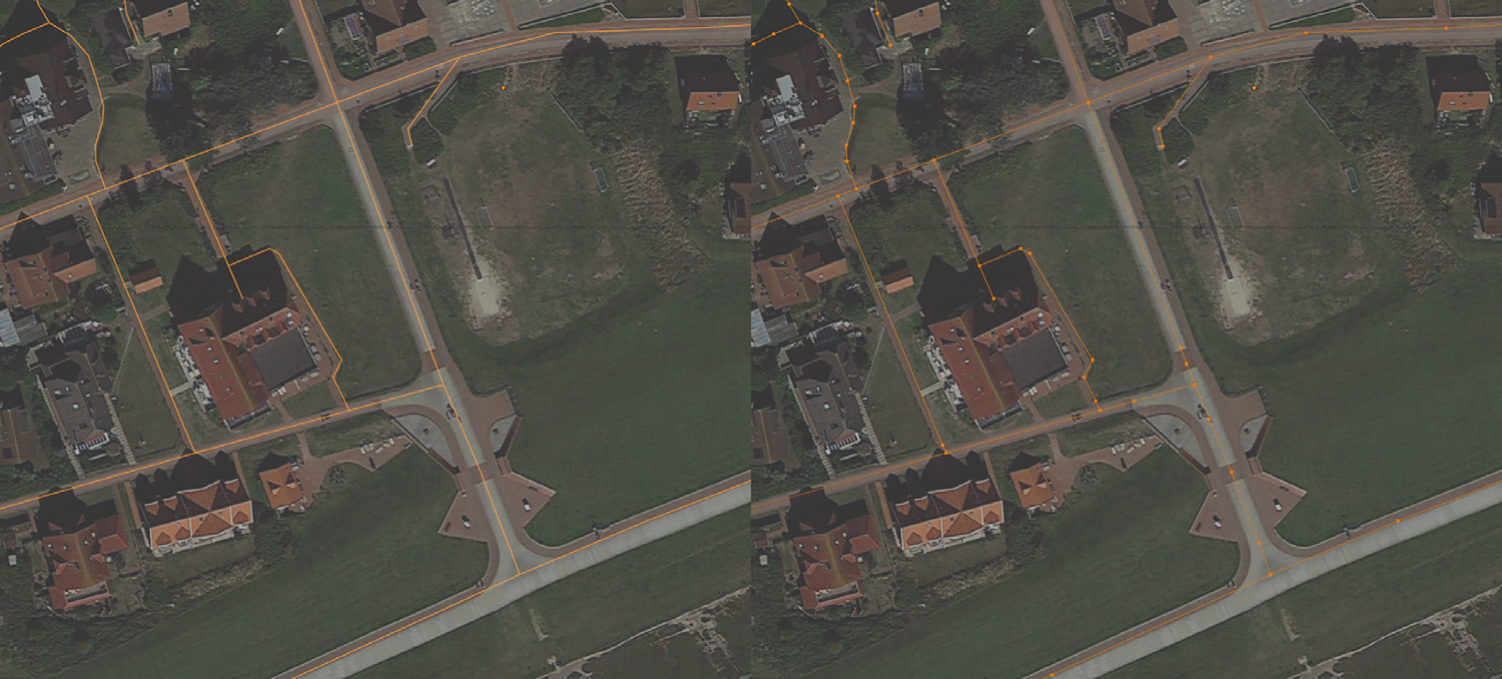
Roads on a map. The left side shows roads colored red and the right side shows roads with vertices that build the roads colored red.

3D models of cars were downloaded as a pack of generic passenger cars from Sketchfab and are available at [3]. Each car 3D model was edited so that its front side is pointing in the positive x-axis direction of the Blender scene. Materials of cars were also edited to have uniform material names, which makes changing the material properties programmatically easier. Cars were then exported as separate FBX (*Filmbox*) files. The map prepared in the previous step contains information about the roads. Roads are made of 3D points - vertices, and lines that connect those vertices - edges. Image 1. shows road data on a map.

Cars were imported one by one. After each car import, the car's location and rotation were modified. A random edge of the road was selected, and its corresponding two vertices were used to calculate the equation of the line that fits the edge. Rotation of the car that would be placed on this edge is equal to the line angle or angle + 180° if the car is going in the opposite direction. After finding the car rotation, its location was decided by choosing a random point on the calculated line. After translating the car to the selected location, the program passes through all the cars previously imported and checks if any of their bounding boxes overlap the bounding box of the newly added car. If that is the case, the newly added car is in collision with another car and to avoid that, a new location for that car is chosen.

After positioning the car, its material was edited. A list of the most common car colors was used [4] to account for the common distribution of car colors. A random color, which would become the base color of the material, was selected from the weighted list of colors.

### 4.3 Adding a camera and Sun to the scene

A Camera object was added to the scene and a list of camera locations was created. Locations were chosen randomly from the map size range. Since this dataset represents images taken from satellites, the camera was set pointing at a nadir direction - camera lens axis perpendicular to the ground.

A Sun object was also added to the scene. Blender has an option to modify Sun position properties. By changing the location attribute of the Sun properties to match longitude and latitude metadata of the georeferenced scene, the location of the Sun copies the location in the real world. This makes the angle and length of the car shadows match the shadows visible on the map.

### 4.4 Rendering images without shadows and effects

Since the map is shaded with emission shader, background images were rendered as they are, with no shadows showing. This step simply renders an image for each camera location and is the first layer rendered.

### 4.5 Adding overlay layer with blurred cars and shadow rendering

**Image 2:**
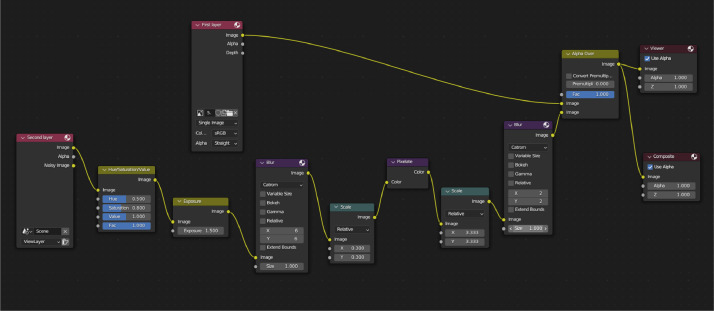
Compositor nodes. Alpha over joins the first layer (background image) and second layer (shadows and edited cars).

A second rendering layer was created with shadows and cars that are blurred. Since the map was shaded with an emission shader, it does not show any shadows on its surface. To get the image of cars with shadows, a shadow catcher plane was created. It is invisible when rendering, but it still takes light and makes shadows visible. To make the second layer blend in with the background layer, blurring was added, and the color was modified. By analyzing maps taken from Google Maps, it was noticed that most of the areas look like they are over exposed and under saturated, so these values are modified for this layer. The second layer is overlaid over the corresponding image of the first layer using the compositor, and the final image is saved. This step is repeated for each camera location. Compositor is, like any other step in this pipeline, edited using Python, but its graphical example is shown on Image 2.

### 4.6 Writing annotations

**Image 3:**
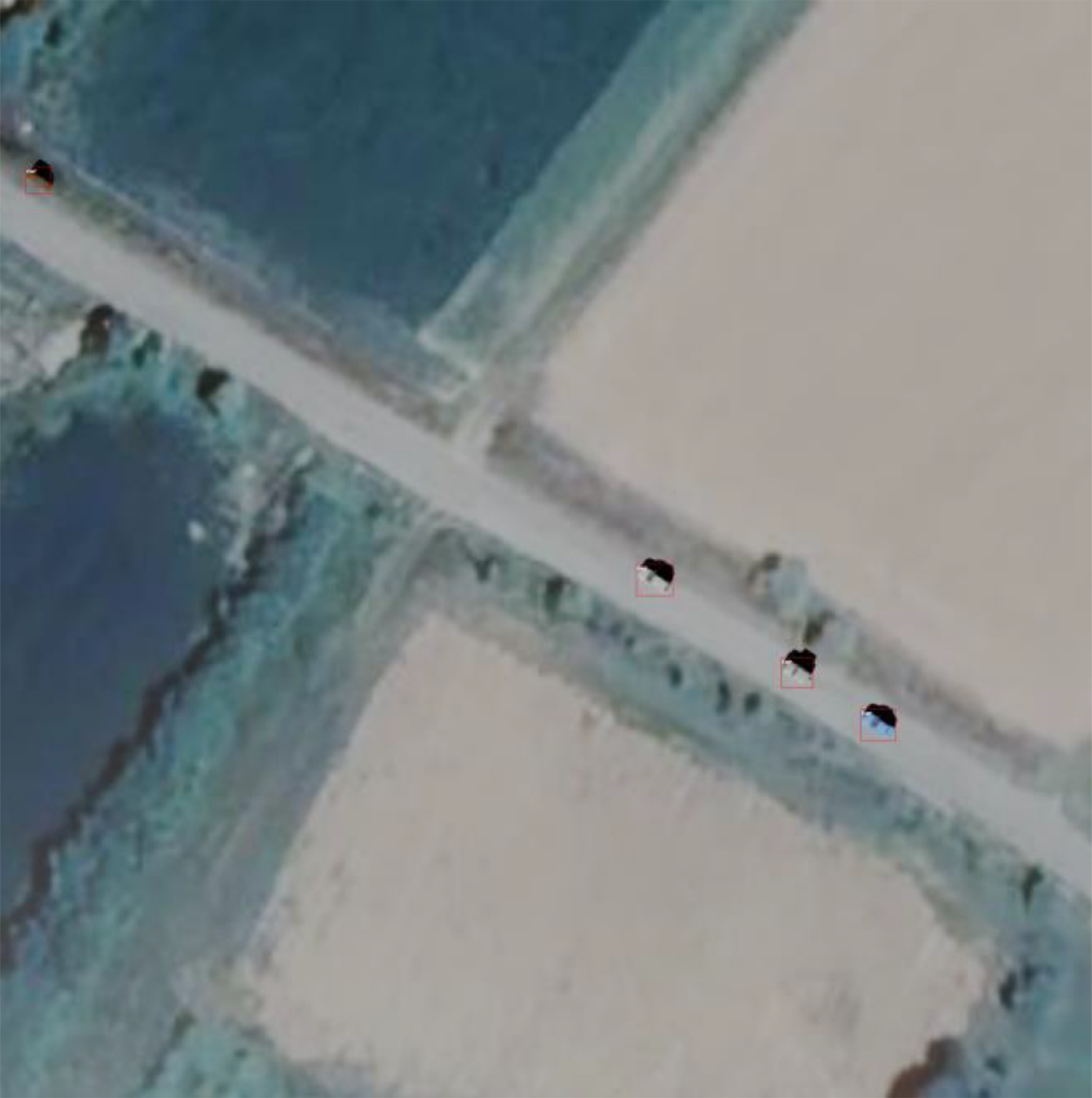
Annotated rendered image with bounding boxes visualized.

Each car on the scene has its rotated bounding box. For each rendered image, box coordinates were transformed to camera space and only those visible in the current image were kept. Bounding boxes were transformed to standard, non-rotated coco format and were saved in JSON format. They were also transformed to fit the YOLO format and were saved as text files. Since Blender originally gives rotated bounding boxes of objects, it is possible to modify this pipeline to save rotated bounding boxes instead of performing the calculations for standard bounding boxes. An example of a rendered image with bounding boxes visualized is shown on Image 3.

### 4.7 Optional step - other vehicles are added

This dataset has one class - car, but the structure of the pipeline that created the dataset allows easy adding of new classes. Other vehicle classes would be more specific and more unique visually in contrast to cars. The range of dimensions of all car types is limited. Furthermore, shapes of different car types do not look too different from large distances. This made it possible to collect a set of 3D models that would cover the whole representation of various car types. On the other hand, for the creation of the dataset with other vehicles, such as farms, military, heavy transport vehicles, etc., special 3D models would have to be found or even created.

The dataset was created using a PC with AMD Ryzen Threadripper 3970X processors, 125GB of RAM and two Nvidia Titan RTX graphics cards. Rendering engine used in Blender was Cycles with a maximum sample size of 64. All of the settings for creation are in a config file and can be easily modified to change render quality and dataset size.

## Limitations

Our current dataset encompasses a limited selection of only 10 3D car models, a small fraction compared to the extensive variety available in global markets. This limitation is particularly relevant for tasks requiring detailed classification, as the dataset's narrow scope may not adequately represent the wide range of existing vehicle types. However, this constraint does not diminish the utility of our pipeline. In fact, our methodology is designed to be adaptable, allowing for the creation of synthetic datasets tailored for both the detection and classification of a broader array of known vehicles within the intended context.

Additionally, we acknowledge a limitation in the resolution of our orthomosaics, which is bound by the quality of available imagery. Despite this, our pipeline is versatile and can be expanded to incorporate high-resolution images, enhancing the detail and accuracy of our models. This adaptability ensures that our approach remains relevant and effective, even as the demands for higher resolution and more diverse vehicle classification grow.

## Ethics Statement

Authors confirm that they have read and follow the ethical requirements for publication in Data in Brief and confirm that the current work does not involve human subjects, animal experiments, or any data collected from social media platforms.

## CRediT authorship contribution statement

**Mihaela Orić:** Data curation, Formal analysis, Investigation, Methodology, Software, Writing – original draft, Writing – review & editing. **Vlatko Galić:** Methodology, Supervision, Writing – original draft. **Filip Novoselnik:** Conceptualization, Methodology, Resources, Supervision, Writing – original draft.

## Figures and Tables

**Image 4:**
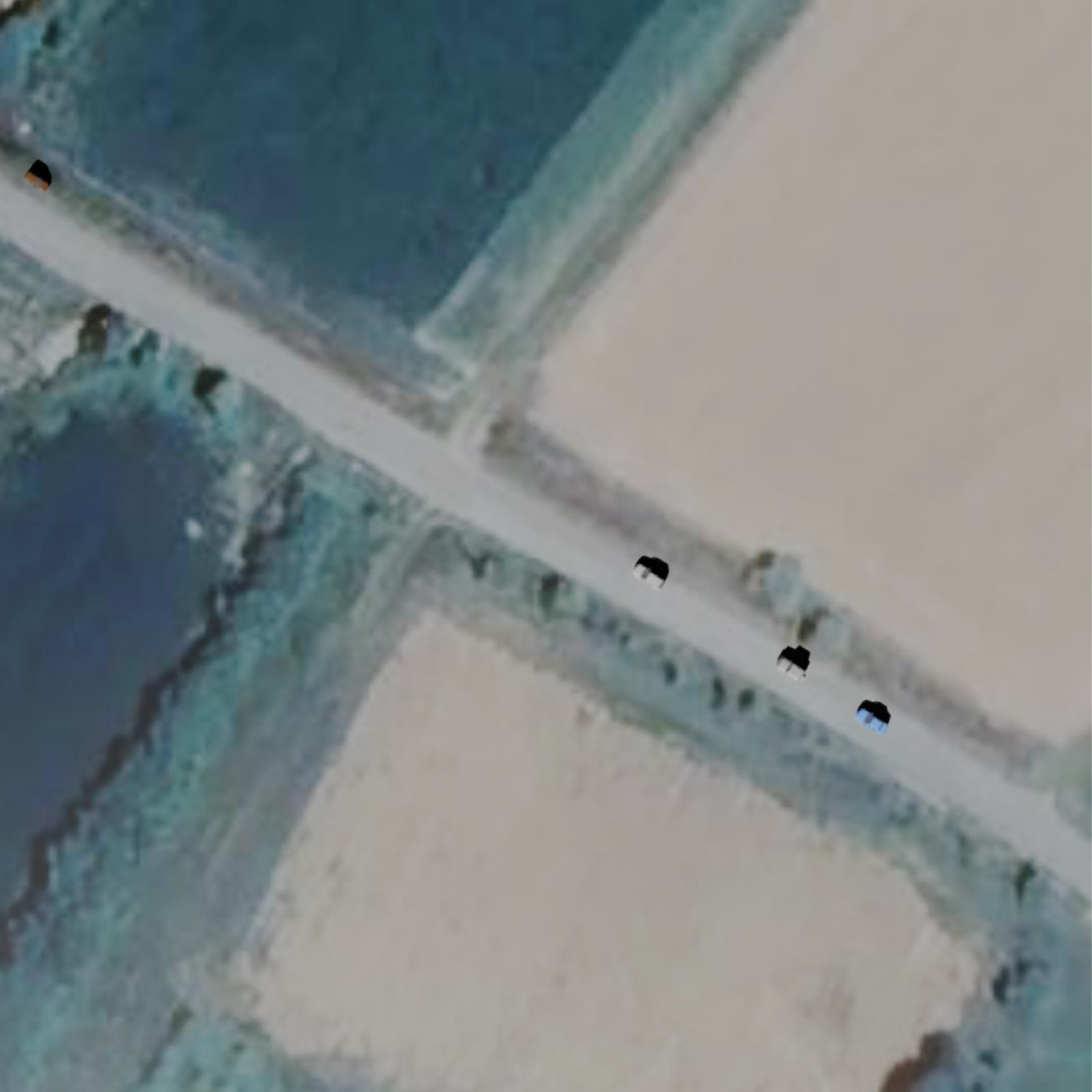
Example of cars placed on a road.

**Image 5:**
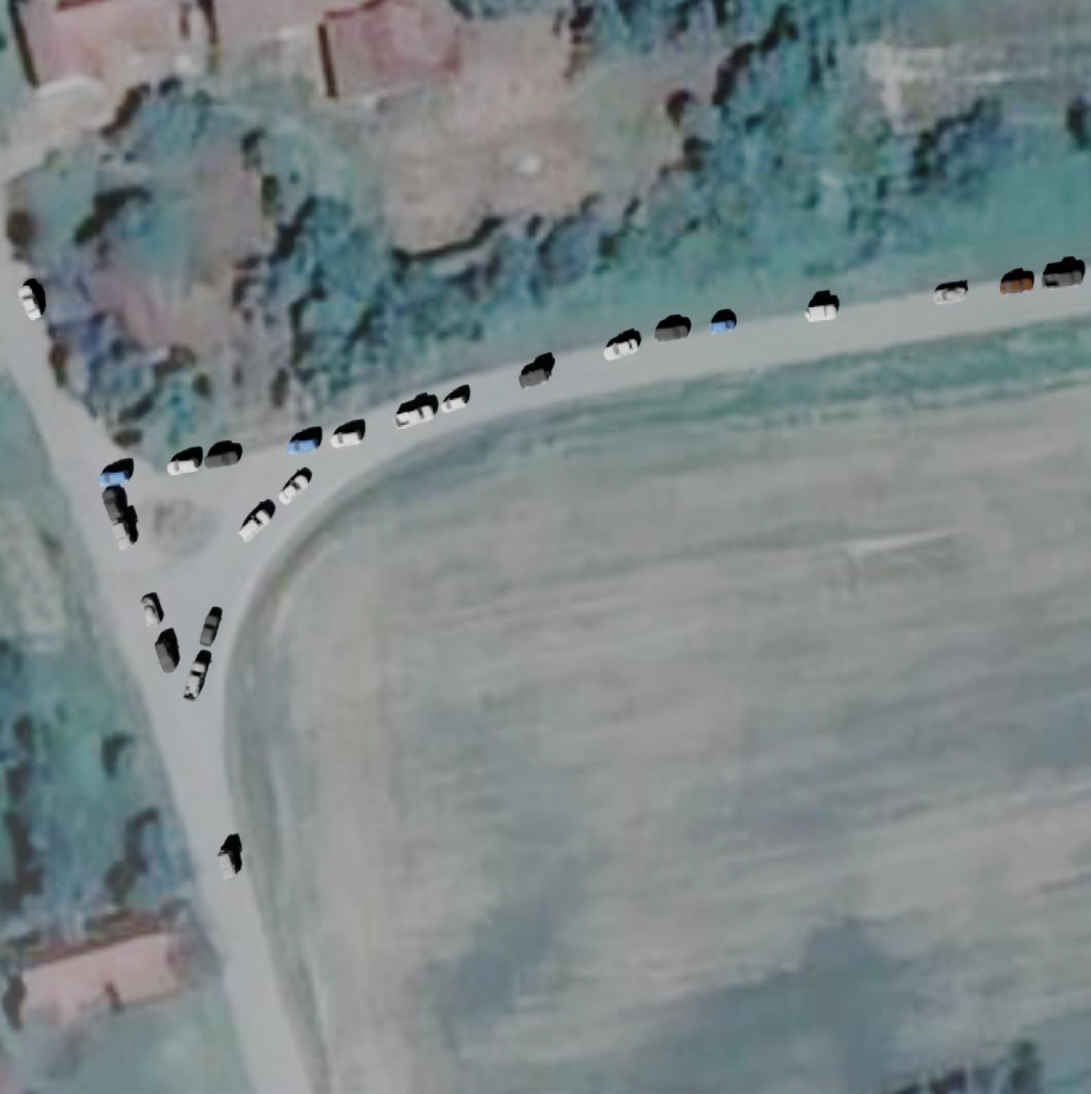
Example of cars placed on roads.

**Image 6:**
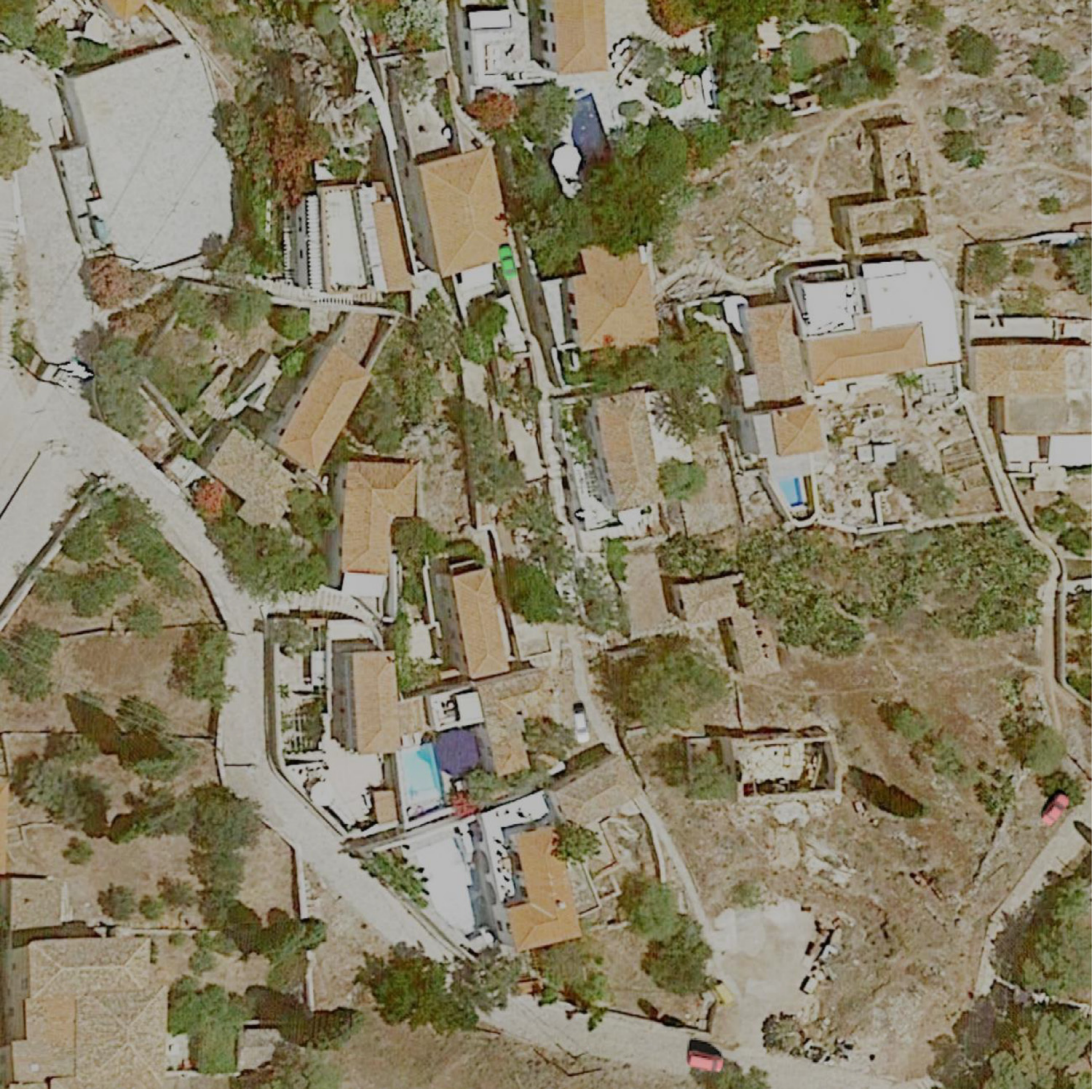
Example of cars placed on roads.

**Image 7:**
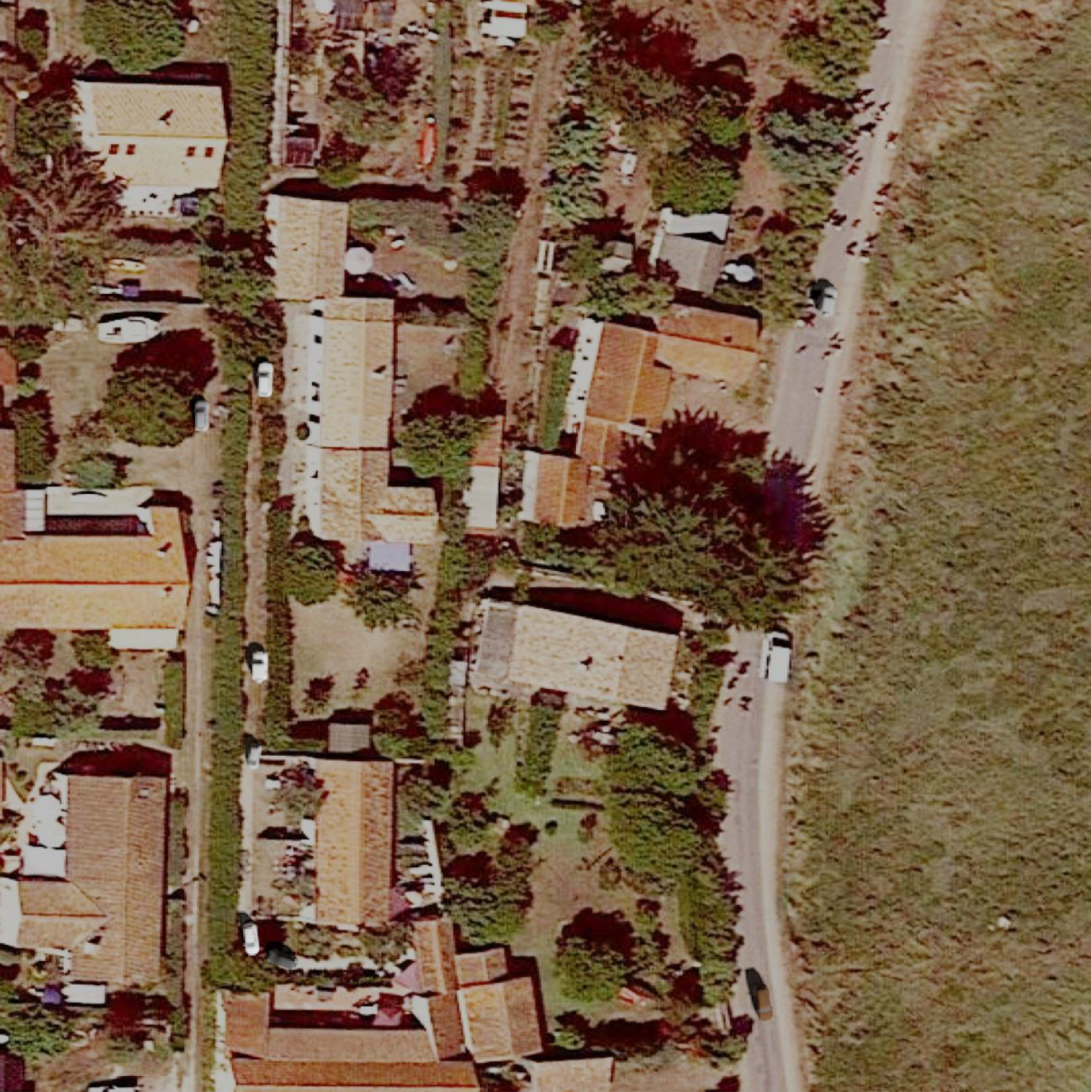
Example of cars placed on roads.

**Image 8:**
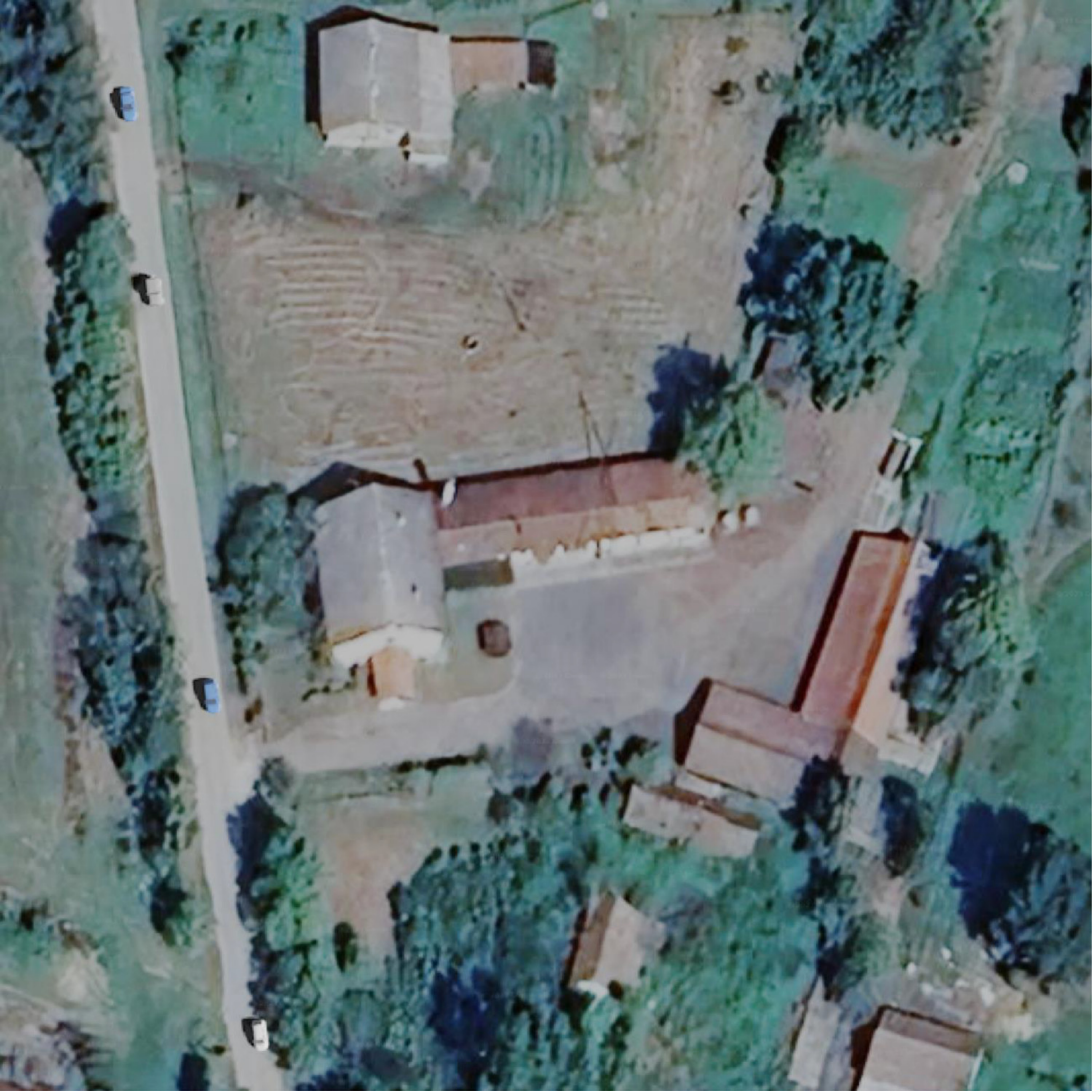
Example of cars placed on a road.

**Image 9:**
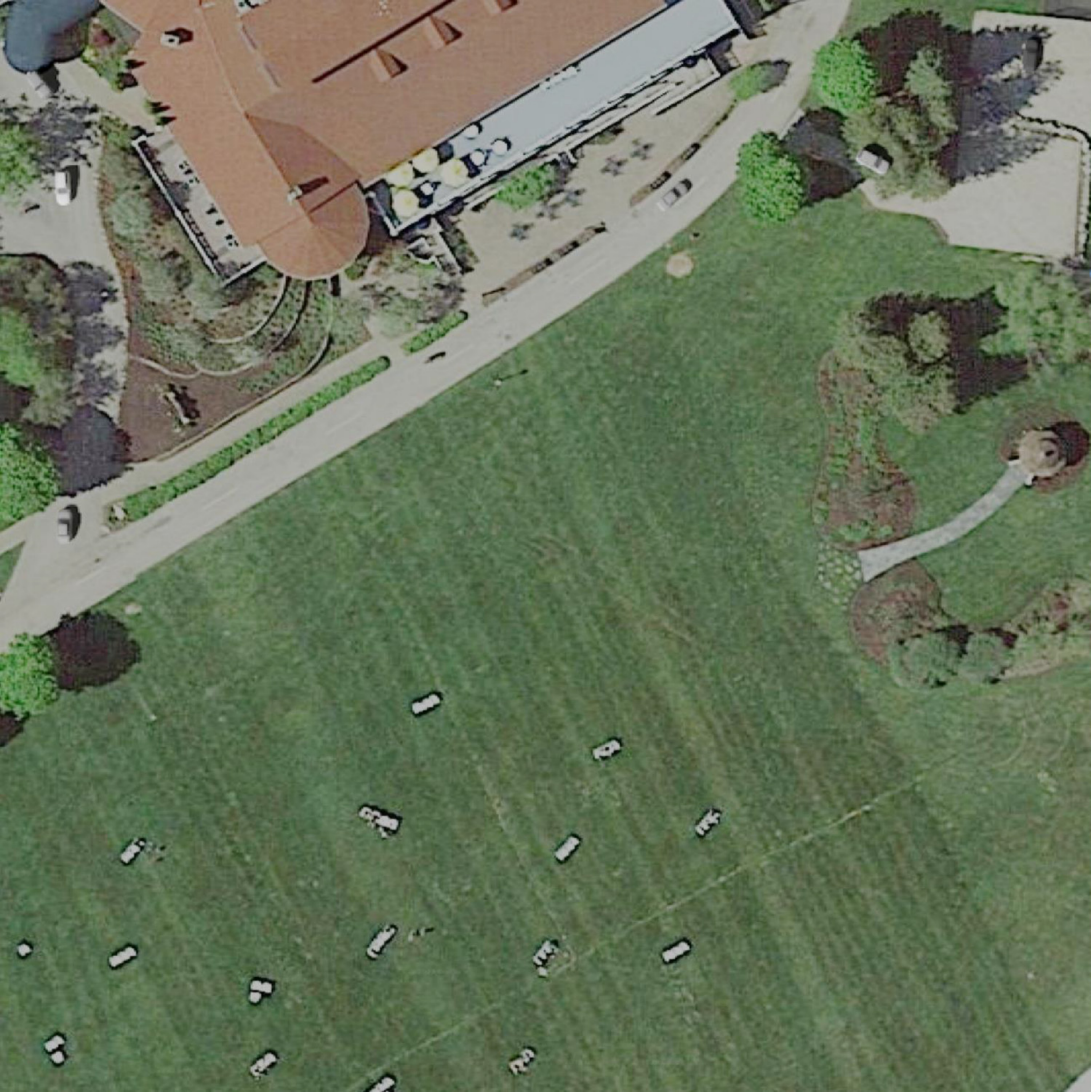
Example of cars placed on roads.

**Image 10:**
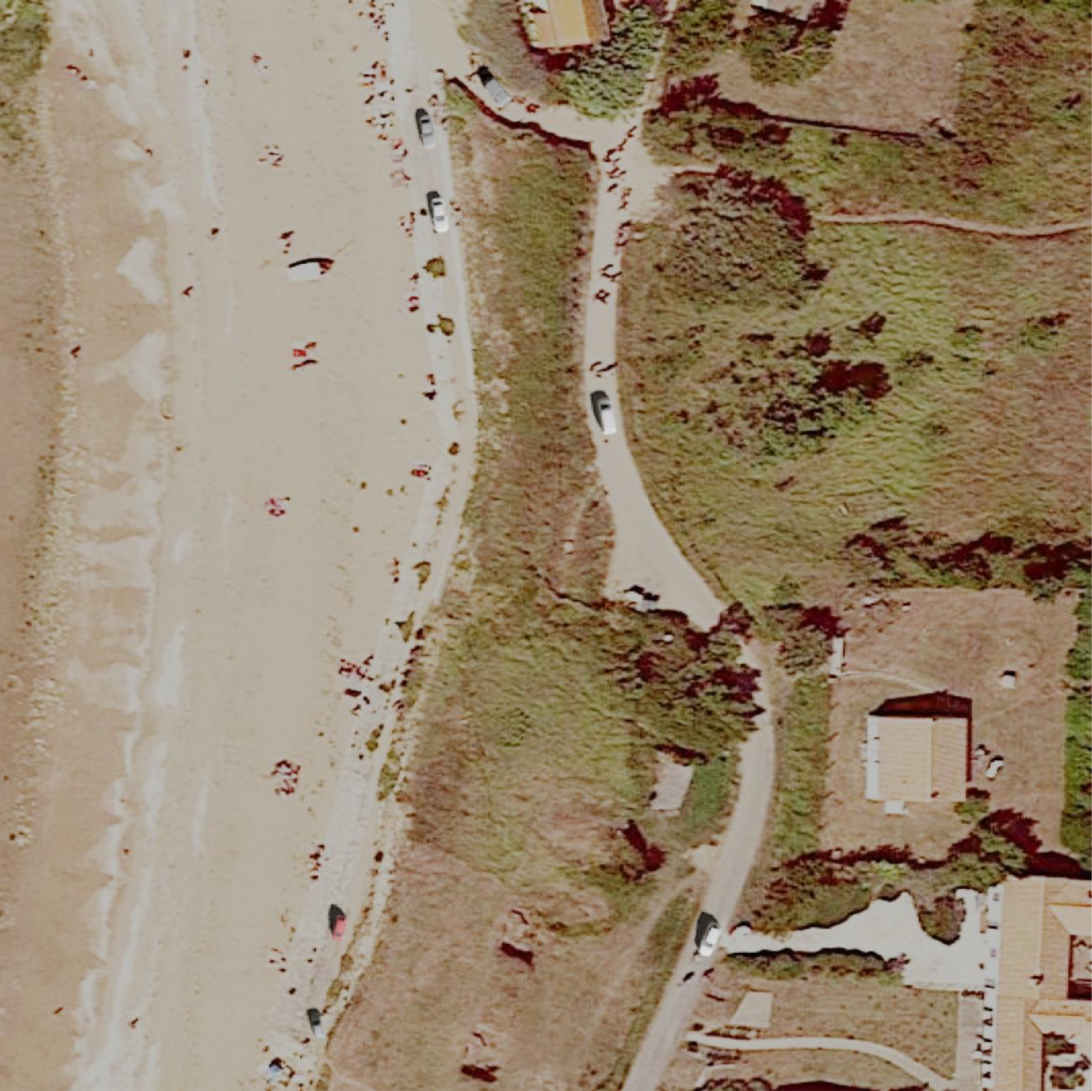
Example of cars placed on roads.

**Image 11:**
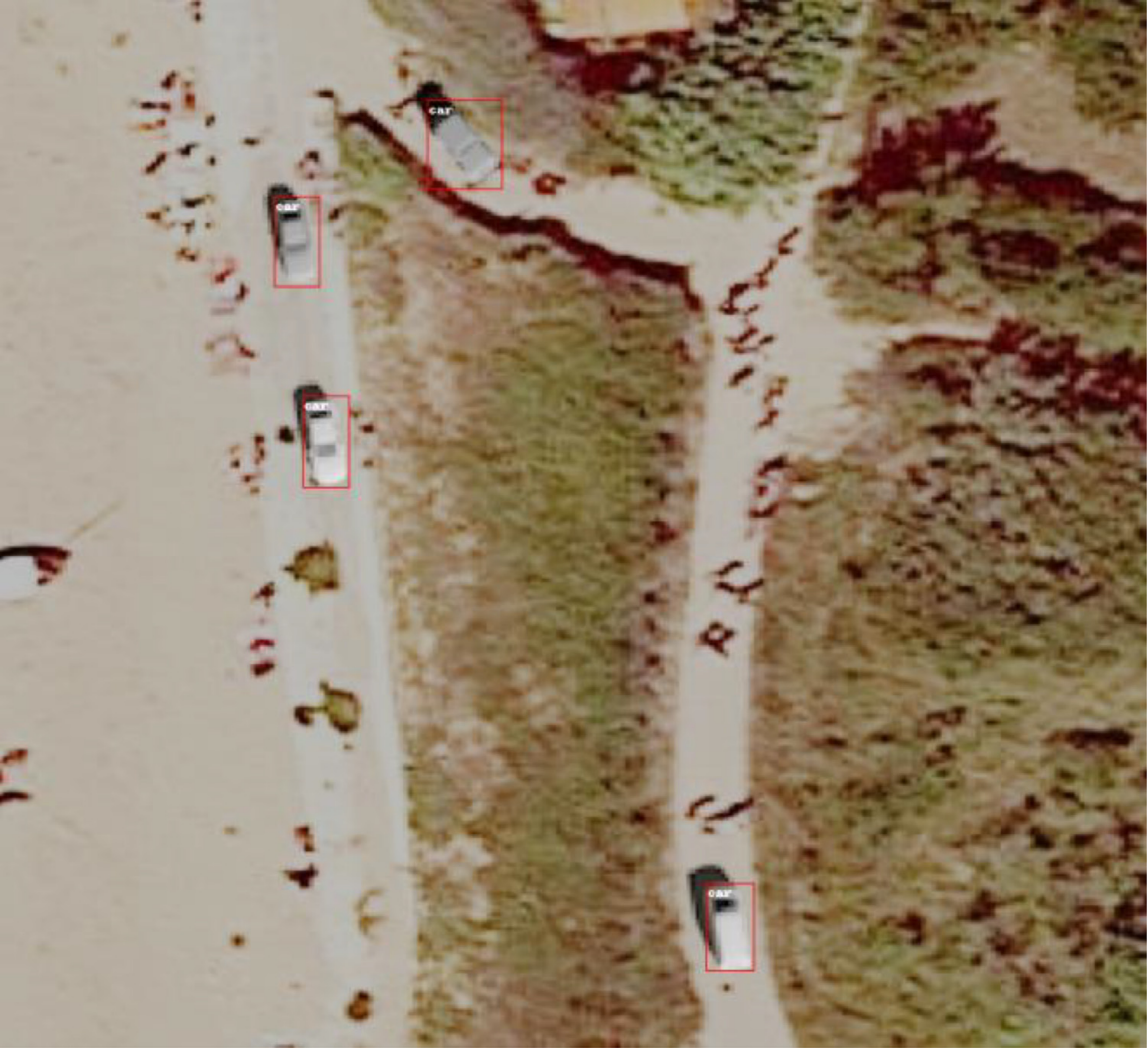
Example of cars placed on roads with their bounding boxes visualized.

**Image 12:**
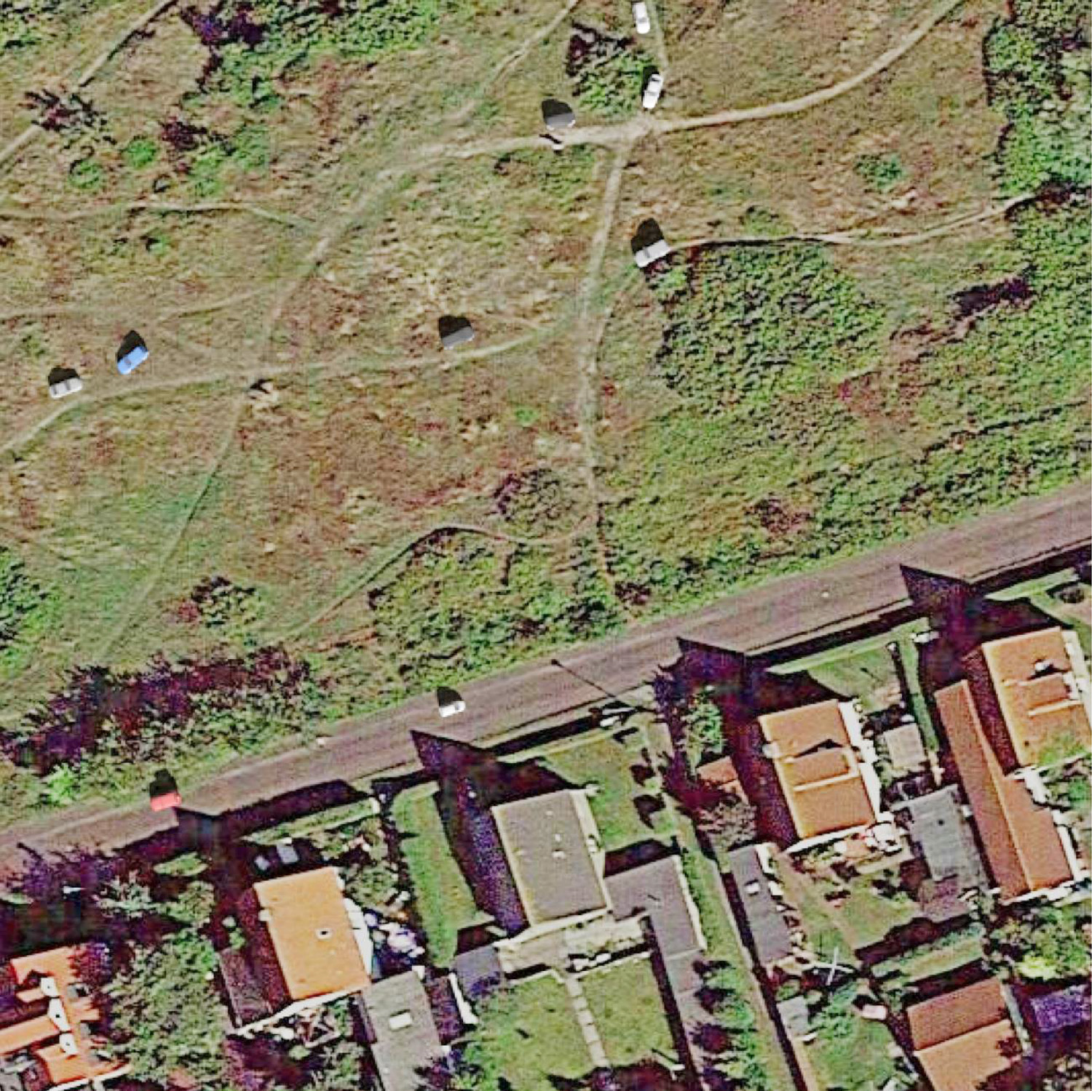
Example of cars placed on roads.

**Image 13:**
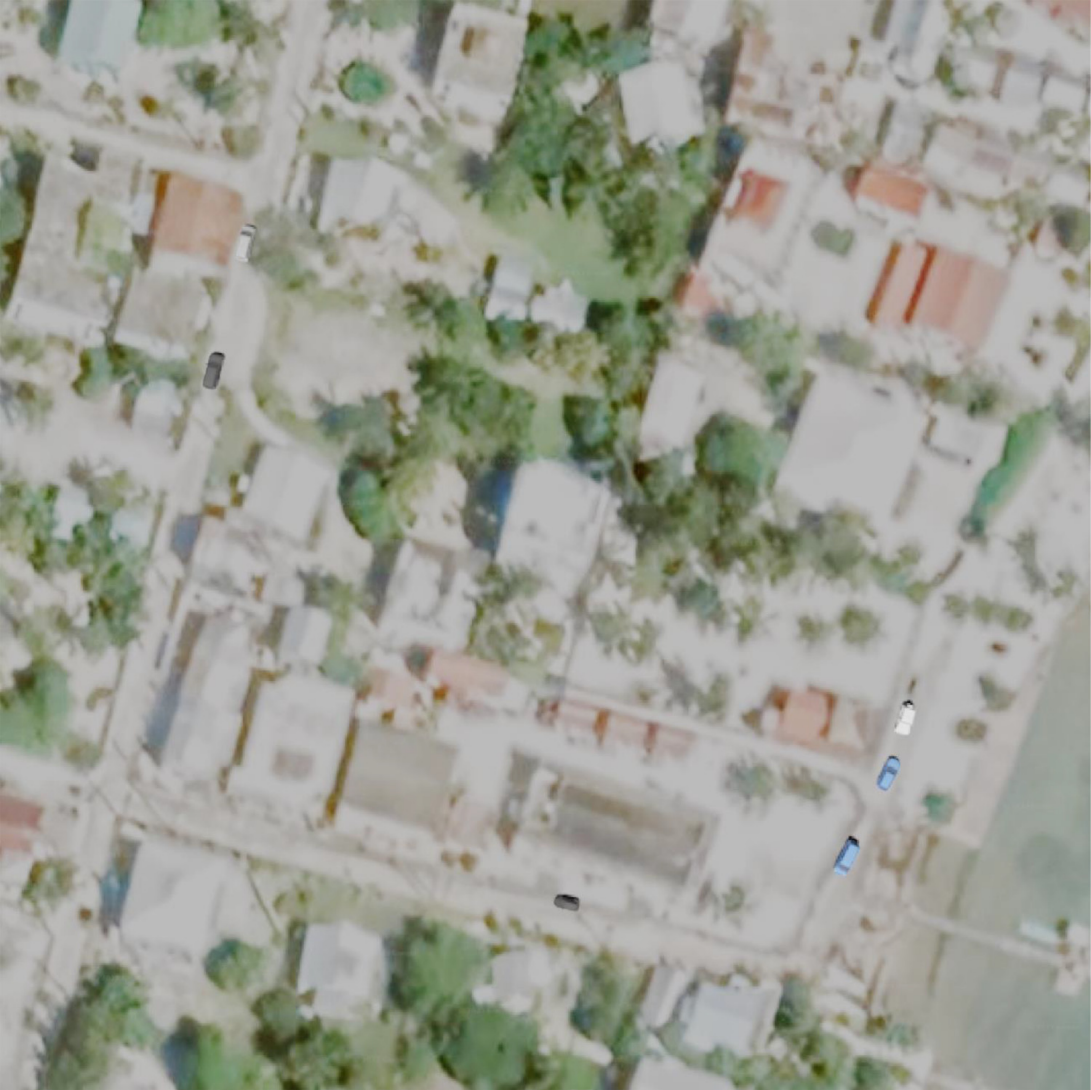
Example of cars placed on roads.

**Image 14:**
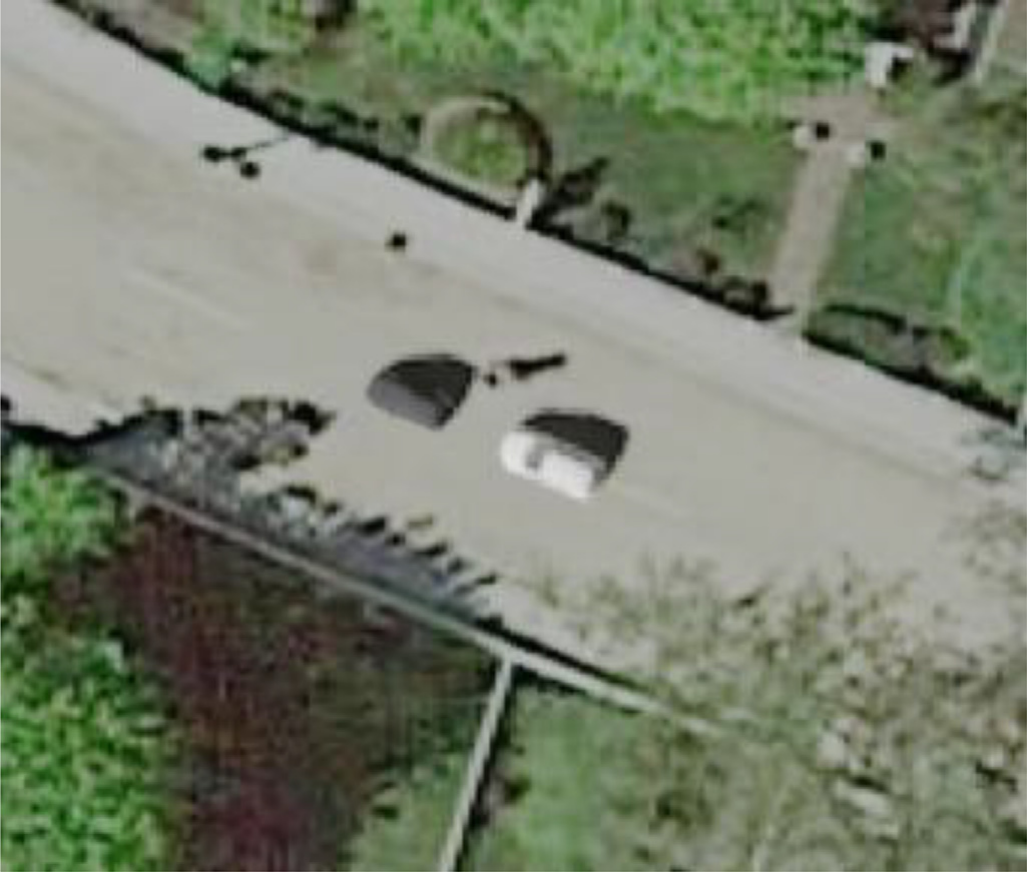
Example of cars placed on a road.

**Image 15:**
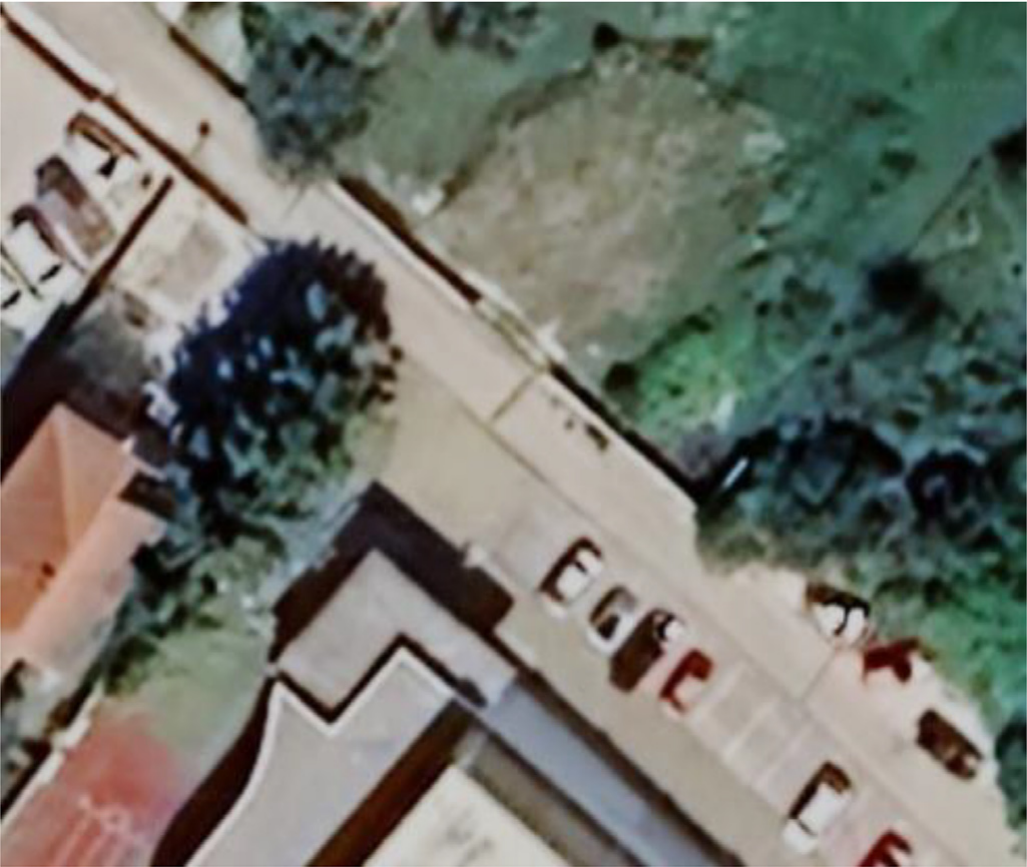
Example of cars placed on roads.

## Data Availability

Synthetic Car Dataset (Original data) (Zenodo)

## Examples from the dataset

Image 4, Image 5, Image 6, Image 7, Image 8, Image 9, Image 10, Image 11, Image 12, Image 13, Image 14, Image 15
